# Effect of Adding Yeast Cultures to High-Grain Conditions on Production Performance, Rumen Fermentation Profile, Microbial Abundance, and Immunity in Goats

**DOI:** 10.3390/ani14121799

**Published:** 2024-06-16

**Authors:** Pei Qi, Lizhi Wang

**Affiliations:** Institute of Animal Nutrition, Sichuan Agricultural University, Chengdu 611130, China; qipei@stu.sicau.edu.cn

**Keywords:** yeast culture, high-grain diet, immunity, ruminal fermentation, microbial abundance

## Abstract

**Simple Summary:**

In China, high-grain diets for ruminants due to forage scarcity cause metabolic disorders. Yeast culture promotes rumen health by stabilizing microbial composition and enhancing nutrition and immunity. Therefore, the objective of this experiment was mainly to investigate the effect of the addition of two different species of yeast culture on goat production performance, rumen fermentation profile, microbial balance, and immunity under high-grain conditions. The results indicated that the incorporation of yeast culture into a high-grain diet significantly enhanced the production performance of goats, augmented their immunity, and stabilized the rumen environment. However, different types of yeast cultures acted by different mechanisms. The results provide a reference for the rational application of yeast culture in production.

**Abstract:**

It is a common practice among farmers to utilize high-grain diets with the intention of promoting ruminant growth. However, this approach bears the risk of inducing rumen disorders and nutrient metabolism diseases. Yeast culture (YC) showed advantages in ruminant applications. The objective of this study was to evaluate the effects of adding two different types of YC to high-grain conditions on production performance, rumen fermentation profile, microbial abundance, and immunity in goats. A total of 30 male goats with similar body condition were randomly distributed into 3 dietary treatments with 10 replicates per treatment as follows: basic diet group (CON); basic diet + 0.5% yeast culture 1 (YC1) group; basic diet + 0.5% yeast culture 2 (YC2) group. The trial lasted for 36 days. The results demonstrated that dietary YC supplementation led to an increase in the average daily gain and a reduction in feed intake and weight gain ratio in goats. It increased the apparent digestibility of crude protein, NDF, and ADF (*p <* 0.05). The serum concentrations of interleukin (IL)-1β, IL-6, and Tumor Necrosis Factor-α in the control group were significantly higher than those of the YC groups (*p <* 0.05). The serum concentrations of Immunoglobulin (Ig)A and IgG in the control group were significantly lower than those in the YC groups (*p <* 0.05). The rumen concentration of microbial protein (MCP) in the control group was significantly lower than that in the YC groups (*p <* 0.05). There was a negative correlation between the concentration of IL-10 and *Bacteroidota*, *Spirochaetota*, and *Succinivibrio*, while there was a positive correlation between concentrations of IL-10 and Firmicutes. Nevertheless, discrepancies were observed in the impact of the two different types of YC on the physiological and biochemical indicators of the animals. The concentration of triglyceride in the YC1 group was significantly higher than that of the CON and YC2 groups, while the concentration of urea in the YC2 group was significantly higher than that of the CON and YC1 groups (*p <* 0.05). At the phylum level, the addition of YC2 to the diet significantly increased the relative abundance of Bacteroidota and Fibrobacterota and significantly decreased Firmicutes compared to the control. At the genus level, the addition of YC1 to the HGD significantly reduced the relative abundance of *Rikenellaceae_RC9_gut_group*, while the addition of YC2 to the HGD significantly increased the relative abundance of *Prevotellace-ae_UCG-001*, *Fibrobacter*, and *Prevotellaceae_UCG-003* (*p <* 0.05). The addition of YC significantly improved growth performance, increased nutrient digestibility, beneficially manipulated ruminal fermentation and microbial diversity, and improved immune function. The choice of yeast cultures can be customized according to specific production conditions.

## 1. Introduction

In China, due to the severe shortage of high-quality forage, it has become increasingly common to feed ruminants high-grain diets (HGD) to ensure high growth performance [1]. However, the concentration of harmful substances such as lipopolysaccharide (LPS) and histamine (His) in the rumen increases significantly when goats are fed HGD for prolonged periods. In addition, an imbalance in the rumen bacterial community may occur, leading to a significant reduction in the diversity of the rumen microflora [2,3,4]. The rumen is a unique digestive organ of ruminants, which contains many anaerobic microorganisms, including fungi, bacteria, and protozoa [5]. The growth of ruminants is dependent on the symbiotic relationship between the host and the rumen microorganisms, which provide the host with energy, protein, vitamins, and other essential nutrients [6]. The microbial community composition of the digestive tract in ruminants is influenced by various factors, including species, age, environment, and diet [7,8,9,10,11]. Of these factors, diet has the greatest impact on rumen microorganisms [12,13]. The composition of rumen microorganisms not only affects the digestion and absorption of the host’s diet but also has a significant impact on the host’s health and immune function. Therefore, maintaining rumen environment homeostasis, improving animal immunity, and reducing inflammatory reactions have become hot topics under the condition of HGD feeding. Yeast culture (YC), as a prebiotic micro-ecological product, consists of a small number of residual yeast cells, yeast metabolites, yeast cell wall fragments, and part of the culture medium [14]. YC contains a wide variety of biologically active substances, including proteins, small peptides, oligosaccharides such as β-glucan and mannan, vitamins, minerals, enzymes, and numerous ‘unknown growth factors’, which have beneficial nutritional and health effects on animals [15]. Probiotics in yeast cultures can regulate the structure of the rumen microbial community, inhibit the growth of lactic acid-producing bacteria such as Lactobacillus, reduce the accumulation of lactic acid, maintain the acid-base balance in the rumen, and reduce the adverse effects on animals [16,17]. The active ingredients in yeast cultures have been demonstrated to stimulate the animal’s immune system, promote the production and secretion of antibodies, and enhance the animal’s resistance to disease [18]. Although numerous studies have been conducted on the role of YC in ruminants, there are limited studies on the effects of yeast culture added into HGD [19,20,21,22]. Meanwhile, the effects of different types and processes of YC may vary significantly. Therefore, we hypothesized that YC may influence rumen fermentation patterns by affecting the abundance of major functional flora, therefore mitigating the adverse effects of prolonged feeding of high-grain diets. This experiment was conducted to evaluate the effects of YC on the production performance, ruminal fermentation characteristics, rumen bacterial populations, and immunity in goats fed with HGD.

## 2. Materials and Methods

The research protocol for this study was approved by the Animal Policy and Welfare Committee of the Agricultural Research Organization of Sichuan Province, Chengdu, China (approval code: SCAUAC201408-3), and it adheres to the guidelines of the Animal Care and Ethical Committee of Sichuan Agricultural University.

### 2.1. Experimental, Animals, Design, and Diet

The experiment took place at Sichuan Agricultural University’s Animal Nutrition Institute’s experimental farm in Ya’an, China. A total of 30 healthy fattening goats (6 months of age, male, vaccinated and dewormed, uncastrated, BW = 25.63 ± 0.36 kg) with similar body condition were randomly distributed into 3 dietary treatments (10 replicates/treatment) as follows: Basic diet group (CON); basic diet + 0.5% yeast culture 1 (YC1) group; basic diet + 0.5% yeast culture 2 (YC2) group. The animals were both vaccinated and dewormed. The YC1 was supplied by Bio-Nutrition International Company (Madison, MI, USA), and the YC2 was supplied by Vitech Ultra Bioscience Corporation (Orange, CA, USA). The main components of YC1 include mannan, dextran, proteins, peptides, amino acids, organic acids, vitamins, mineral salts, nucleotides, active peptides, esters, and alcohols. The main components of YC2 include mannan-oligosaccharides, beta-glucan, surfactants, proteins, peptides, amino acids, organic acids, vitamins, mineral salts, and nucleotides. Following the guidelines of the Chinese Feeding Standard for Meat-producing Sheep and Goats [23], the experimental diet was designed, as detailed in Table 1. Goats received a Total Mixed Ration (TMR). Each goat was kept separately in metabolic crates that included feeders and water containers. The experimental diet was provided twice daily at approximately 9:00 a.m. and 5:00 p.m. for ad libitum intake. During the pre-feeding period, the quantity of feed and the quantity of leftovers were recorded daily for each test sheep. The quantity of the following day’s feed was then adjusted according to the quantity of leftovers from the previous day, with the objective of ensuring that approximately 5% of the feed remained in the trough daily. Fresh water was continuously available. After an acclimation period of 10 days, the formal trial lasted 36 days, consisting of a 30-day feeding phase and a 6-day digestion phase.

### 2.2. Product Performance

During the treatment period, the initial individual and final body weight (BW) at the beginning of the feeding experiment were recorded. A daily record of the amount of feed consumed was kept, and the average daily feed intake (ADFI), average daily gain (ADG), and feed-to-gain ratio (F/G) were calculated.

### 2.3. Sample Collection of Blood and Ruminal Fluid

Collection of feed samples: Appropriate samples were collected from the diets to be tested using the tetrad method, and the feed samples collected in each period were mixed and stored using a sealing bag for nutrient determination.

Collection of fecal samples: During the digestion test period, all fecal matter from each sheep was collected and weighed at approximately 09:00 and 17:00 to record the weight. Every day, the feces were mixed and weighed to take 10% of them. One part of them was added to sulfuric acid for nitrogen fixation. The method of addition was to add 10% sulfuric acid (10 mL per 100 g of fresh feces) to avoid the loss of ammonia nitrogen in the feces. The samples were preserved at −20 °C for the determination of fecal nitrogen. The other part was used for air-dried sample preparation and determination of nutrient composition. At the conclusion of the experiment, the freshly collected manure samples were mixed with sulfuric acid and then stored at −20 °C until the determination of manure nitrogen. The remaining manure samples from each day were combined to create air-dried samples for the analysis of dry matter (DM) and other nutrient contents.

Collection of rumen content and blood samples. Rumen content and blood samples were collected at 9:00 a.m. on the 30th day of the experiment, prior to the morning feed. The blood samples were collected from the jugular vein of the goat. The samples were maintained on ice until all had been collected, after which they were processed immediately in the laboratory. Centrifugation at 4000 rpm for 15 min was performed to obtain serum, after which the samples were frozen at −20 °C for the testing index. The rumen content was collected via oral sampling, with the oral collector being inserted into the oral cavity and then into the rumen. The initial rumen content sample was discarded to prevent contamination from reticulum fluid, salivary fluid, or bacteria on the animal’s body surface. A subsequent sample of 300 mL of rumen content was then collected. The rumen content was divided into two portions, one of which was squeezed through four layers of gauze. The rumen fluid was collected, and the pH was tested immediately [24].

### 2.4. Laboratory Analyses

The samples were dried at 55 °C and then analyzed for dry matter content at 105 °C (DM, method 934.01), crude protein (CP, method 984.13), and ether extract (EE, method 973.1) in accordance with the protocols established by the Association of Official Analytical Chemists [25]. The levels of neutral detergent fiber (NDF) and acid detergent fiber (ADF) were quantified using the methodology described by Van Soest et al. [26]. The apparent nutrient digestibility was calculated using the following formula [27,28]:

Apparent digestibility of nutrient (%) = (ingested nutrient − excreted nutrient in feces)/(ingested nutrient) ×100

Metaphosphoric acid (0.25 mL) was added to 1 mL rumen fluid and centrifuged at 15,000× *g* for 15 min, and gas chromatography (GC-MS, Agilent Technologies, Palo Alto, CA, USA) was used to detect acetic, propionic and butyric acid concentrations in the rumen fluid. Serum Immunoglobulin (Ig)G, IgA, IgM, Tumor Necrosis Factor-α(TNF-α), Interleukin (IL)-1β, IL-6, IL-10, and lipopolysaccharide (LPS) were detected by double antibody sandwich ELISA (ELISA kit: Meimian Biotechnology, Yancheng, Jiangsu, China).

The concentrations of glutamic pyruvic transaminase (ALT), glutamic oxaloacetic transaminase (AST), urea creatinine (CREA), alkaline phosphatase (ALP), lactate dehydrogenase (LDH), creatine kinase (CK), total protein (TP), albumin (ALB), total cholesterol (TC), triglyceride (TG), low-density lipoprotein cholesterol (LDL-C), high-density lipoprotein cholesterol (HDL-C) in serum were detected by automatic biochemical analyzer (Hitachi 3100, Hitachi Limited, Tokyo, Japan) using standard kit (Zhongsheng Beiqin Biotechnology Co., Ltd., Beijing, China).

### 2.5. DNA Extraction and Amplification

To extract DNA, 1 mL of rumen fluid was centrifuged at 12,000× *g* for 10 min at 4 °C. The DNA extraction was conducted using the TIANamp Bacteria DNA Kit (Tianjin, China) in accordance with the methodology proposed by Guo [29] and in accordance with the instructions provided by the manufacturer. The purity and concentration of the extracted DNA were assessed via gel electrophoresis. The quality of the extracted bacterial DNA was assessed via agarose electrophoresis and a NanoDrop 8000 spectrophotometer (Thermo Fisher Scientific, Melbourne, Australia). The high-quality DNA was then amplified with bacteria-specific primers targeting the V4 hypervariable region of the 16S rRNA gene. The forward primer was 5′-GTGCCAGCMGCCGCGGTAA-30′ (515F), and the reverse primer was 5′-GGACTACVSGGGTATCTAAT-3′ (806R) [30]. Each sample was assigned a unique 5-8-base error-correcting barcode on the 515F primer for multiplex sequencing. The amplicons were subsequently submitted to Novogene Technology Company (Beijing, China) for sequencing on the MiSeq Illumina Sequencing Platform in accordance with the protocols outlined by Caporaso [31].

### 2.6. Bioinformatics and Statistical Analysis

The reads acquired from Novogene were analyzed using the QIIME pipeline software (version 1.8.0) [32] in accordance with the previously described method. Sequences containing uncertain nucleotides, unmatched barcodes, or three consecutive nucleotides with Q values below 20 were discarded. The Uchime algorithm in QIIME was then employed to eliminate chimeric sequences using USEARCH V7.0 [33]. To reduce the impact of sequencing noise, a pre-clustering methodology was employed [34]. The sequences were then clustered into operational taxonomic units (OTUs) using the Uclust method at 97% similarity, with a representative sequence selected for each OUT [35]. The representative sequences were then aligned against the Greengenes database (http://greengenes.lbl.gov, accessed on 6 December 2023) and assigned taxonomy via the RDP Classifier [36]. Six alpha-diversity indices of the bacterial communities (Chao1, Dominance, goods_coverage, observed_features, pielou_e, and Shannon) were calculated. Beta diversity was visualized using principal coordinate analysis (PCoA), which is based on an unweighted UniFrac distance matrix [37]. The relative abundance of bacterial phyla and genera was depicted using histograms created with OriginPro software (version 9.0). Furthermore, a heatmap generated by the R software (version 3.4.2) was employed to illustrate the genera shared by all samples. Finally, R software was used to analyze the significant species differences between groups.

### 2.7. Correlation between Rumen Microbiota and Blood Parameters Variables

Nonparametric Spearman rank correlation coefficient analysis implemented in SPSS statistical software (Ver. 27.0 for Windows; SPSS Inc., Chicago, IL, USA) was used to analyze the relationship between blood parameters and the relative abundance of rumen bacteria in rumen fluid. The correlation matrix was displayed as a heatmap using the corrplot package in R (Corrplot: visualization of a correlation matrix, R package version 0.2-0, 2010) [38].

### 2.8. Statistical Analysis

All data are presented as the mean ± SE. Each index was analyzed with 10 replicates. Statistical analyses were carried out using the SPSS statistical software (Ver. 27.0 for Windows; SPSS Inc., Chicago, IL, USA). The Shapiro–Wilk test and Levene’s test were performed to test the data for normality and homoscedasticity, respectively. The differences in the relative bacterial abundance among the three groups were analyzed by a nonparametric test, and other parameters were analyzed by one-way analysis of variance (ANOVA) followed by Duncan’s multiple comparisons to determine significant differences among the treatments. Differences were considered significant at *p <* 0.05.

## 3. Results

### 3.1. Product Performance

The results of the growth performance of goats are shown in Table 2. Dietary YC supplementation increased the goat average daily gain (*p <* 0.001) and average daily feed intake (*p =* 0.009) and reduced the goat F/G (*p =* 0.005).

### 3.2. The Digestibility of Nutrients

The digestibility of nutrients is shown in Table 3. The digestibility of DM, ADF, and NDF in the YC1 and YC2 groups was significantly higher than that in the CON group (*p <* 0.05). There was no significant difference between YC1 and YC2 groups. The digestibility of CP in the YC2 group was significantly higher than that in the CON group, and there was no significant difference between the YC1 and the other groups. The digestibility of EE in the YC1 group was significantly higher than that in the YC2 and CON groups (*p =* 0.009), and there was no significant difference between the YC2 and CON groups.

### 3.3. Blood Parameters

The blood parameters of the JV are shown in Table 4. The concentration of urea in the YC2 group was significantly higher than that of the CON and YC1 groups (*p =* 0.005). The concentration of triglyceride in the YC1 group was significantly higher than that of the CON and YC2 groups (*p =* 0.044). The concentration of high-density lipoprotein cholesterol in the YC2 group was significantly higher than that of the YC1 group (*p <* 0.05). The concentrations of IL-1β, IL-6, and TNF-α in the CON group were significantly higher than those of the YC1 and YC2 groups (*p <* 0.001). The concentration of IL-10 in the YC1 group was significantly higher than that observed in the control and YC2 groups (*p <* 0.001). However, no significant difference was observed in the concentration of IL-10 between the control and YC2 groups (*p >* 0.05). The concentrations of IgA and IgG in the CON group were significantly lower than those in the YC1 and YC2 groups (*p <* 0.05). The concentration of IgM in the CON group was significantly lower than that in the YC2 group (*p <* 0.05). However, no significant difference was observed in the concentrations of ALT, AST, CREA, ALP, LDH, CK, TP, ALB, TC, LDL-C, LPS, HIS, MAP, and HSP-70 among groups (*p >* 0.05).

### 3.4. Rumen Fermentation Characteristics

The results of rumen fluid characteristics are presented in Table 5. The concentration of MCP in the CON group was significantly lower than that in the YC1 and YC2 groups (*p =* 0.039). There was no significant difference in rumen pH, the concentration of NH_3_-N, and rumen volatile fatty acid among three different groups (*p >* 0.05).

### 3.5. Statistics of 16SrRNA Sequencing Results of Rumen Microorganisms

#### 3.5.1. OTU Analysis

**Figure 1:**
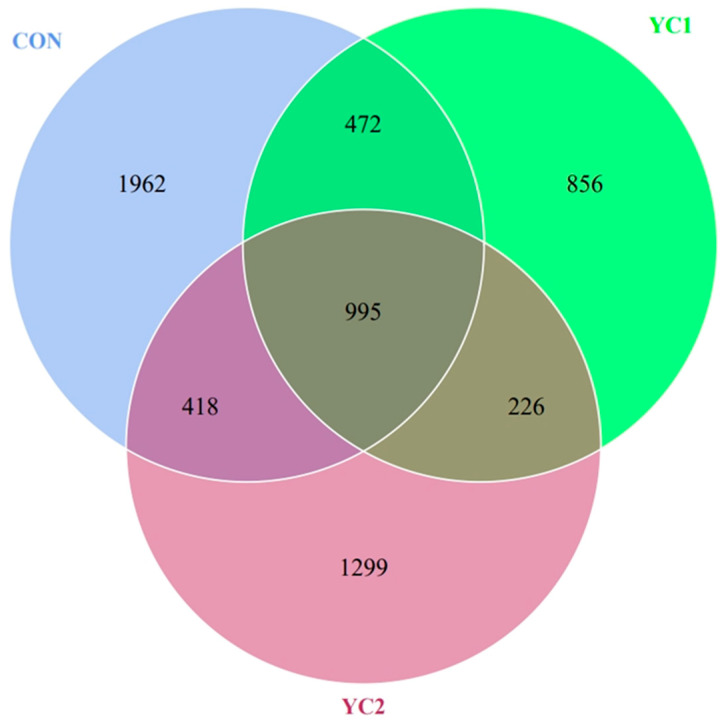
The Venn plot of OTU.

According to the OTUs results and research requirements of noise reduction, the common and unique OTUs among different groups were analyzed. The CON group shared 472 and 418 OTUs, with YC1 and YC2 groups, and the number of shared OTUs among the three groups was 995 (Figure 1).

#### 3.5.2. Alpha-Diversity Analysis

The alpha-diversity indexes estimation of the 16S rRNA gene libraries of the goat rumen that emerged from the sequencing analysis were presented in Table 6. In the current study, the results showed that the chao1 index and observed_otus index in the YC1 group were the lowest and significantly lower than that in the CON and YC2 groups (*p =* 0.002). The pielou_e index, Shannon, and Simpson indexes in the YC1 group were significantly lower than those in the CON group, and there was no significant difference between the YC2 and CON groups (*p >* 0.05).

#### 3.5.3. β Diversity Analysis

**Figure 2:**
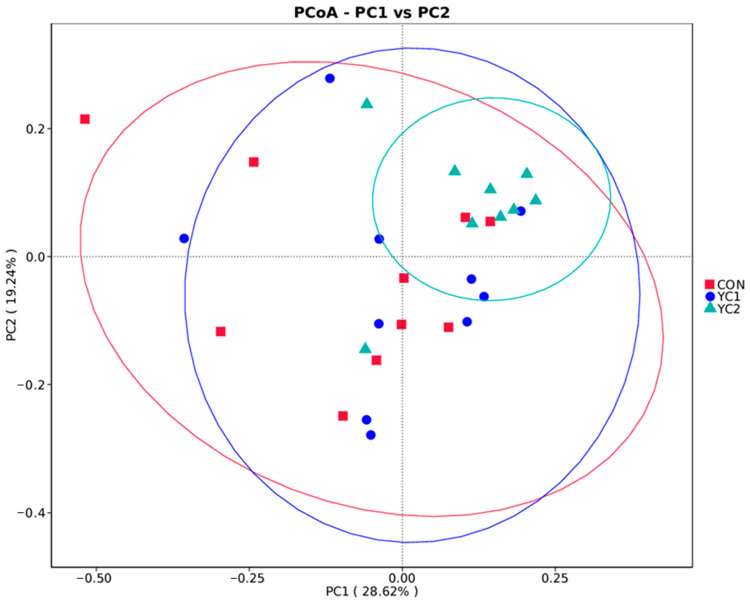
PCoA map of rumen microorganisms.

In the β diversity analysis, weighted UniFrac was selected to measure the difference coefficient between two samples, and PCoA was used to analyze the similarity of each sample. If the sample distance is closer, it indicates that the species composition structure is more similar. As shown in Figure 2, the sample dispersion of the CON group was the highest, while that of the YC2 group was the most concentrated.

#### 3.5.4. Microbiota Composition in Rumen

**Figure 3:**
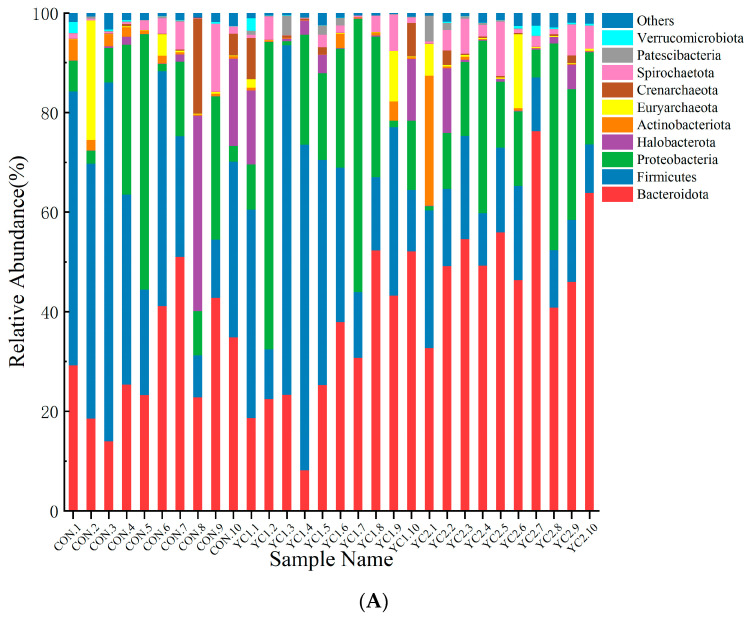

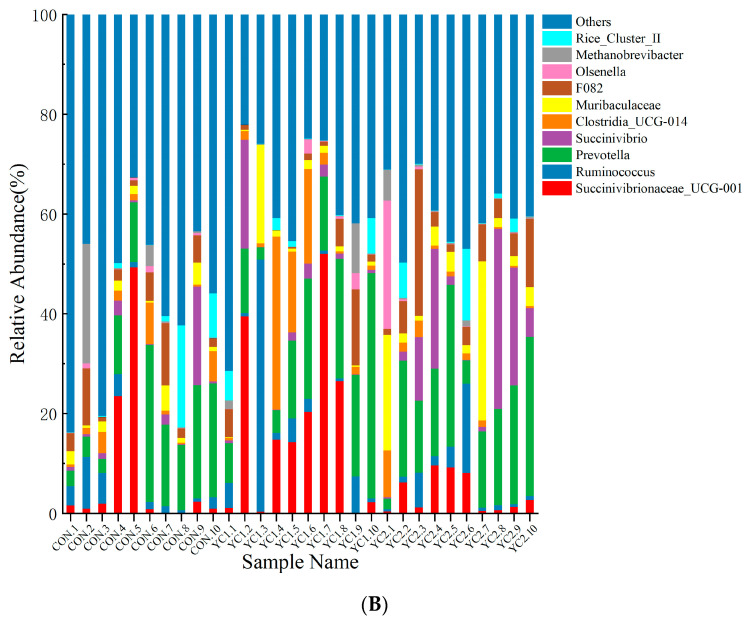
The relative abundance (%) of microbiota in the rumen at the phylum level (**A**) and genus level (**B**). Data represent the relative abundance of the top 10 of the community among the three groups.

At the phylum level, 23 taxa were identified in the rumen. At the genus level, a total of 237 genera were detected in the rumen. Data of the top 10 microorganism populations were analyzed (Figure 3). It was found that a greater relative abundance of Bacteroidota (*p <* 0.05) and Fibrobacterota (*p <* 0.05) in YC2 was higher than in CON (Table 7). Then, the top 10 microorganism populations were analyzed at the genus level. It was found that the relative abundance of *Prevotellaceae_UCG-001* (*p =* 0.006), *Fibrobacter* (*p <* 0.001), and *Prevotellaceae_UCG-003* (*p =* 0.004) in YC2 was higher than in CON and YC1 (Table 7). In contrast, *Prevotellaceae_UCG-010* abundance was lower (*p =* 0.018) in YC1 and YC2 goats compared with the control. In addition, the result showed that *Rikenellaceae_RC9_gut_group* abundance was lower (*p* = 0.012) in YC1 goats compared with control and YC2.

### 3.6. Correlation between Rumen Microbiota and Blood Parameters

**Figure 4:**
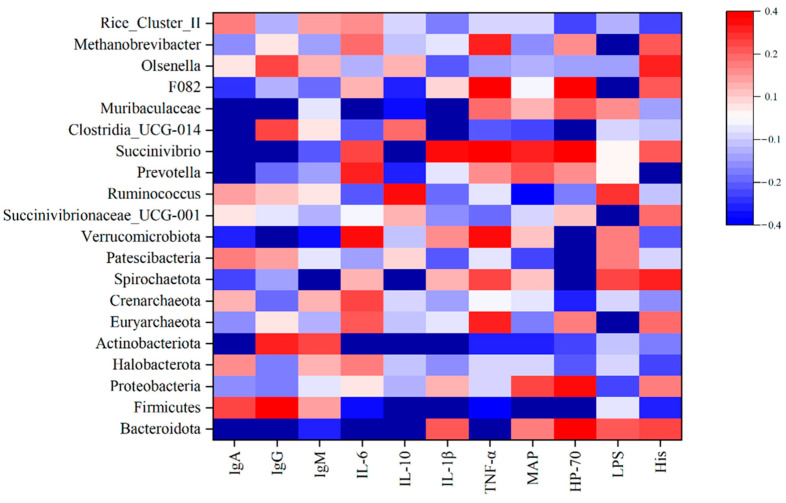
Spearman-parametric rank correlation matrix between blood parameters and microbiota relative abundance (representing at least 0.1% of the bacterial community in at least one sample). The blue color represents a negative correlation, the red color represents a positive correlation, and the white color represents no correlation. Spearman correlations between bacterial and biological parameters at corresponding were analyzed.

The relationship between ruminal microbiota relative abundance (representing at least 0.1% of the bacterial community in at least one sample (in phyla and genus level)) and serum index was analyzed. The results showed (Figure 4) that the concentration of IgA was negatively correlated with Bacteroidota (R = −0.407, *p <* 0.05), *Prevotella* (R = −0.407, *p <* 0.05), *Succinivibrio* (R = −0.374, *p <* 0.01), and Muribaculaceae (R = −0.376, *p <* 0.05), while it was positively correlated with *Clostridia_UCG-014* (R = 0.421, *p <* 0.05). The concentration of IgG was negatively correlated with Bacteroidota (R = −0.406, *p <* 0.05), Verrucomicrobiota (R = −0.558, *p <* 0.01), *Succinivibrio* (R = −0.497, *p <* 0.01), and Muribaculaceae (R = −0.521, *p <* 0.01). The concentration of IgM was negatively correlated with Spirochaetota (R = −0.411, *p <* 0.05) and *Succinivibrio* (R = −0.430, *p <* 0.05). The concentration of IL-6 was negatively correlated with Actinobacteriota (R = −0.479, *p <* 0.01), while it was positively correlated with Bacteroidota (R = 0.458, *p <* 0.05) and Muribaculaceae (R = 0.373, *p <* 0.05). IL-10 was negatively correlated with the relative abundance of Bacteroidota (R = −0.526, *p <* 0.01), Spirochaetota (R = −0.430, *p <* 0.05), and *Succinivibrio* (R = −0.437, *p <* 0.05), while it was positively correlated with Firmicutes (R = 0.484, *p <* 0.01) and Actinobacteriota (R = 0.530, *p <* 0.01). The concentration of IL-1β was negatively correlated with Firmicutes (R = −0.456, *p <* 0.05), Actinobacteriota (R = −0.538, *p <* 0.01), and *ClostridiaUCG-014* (R = −0.478, *p <* 0.01), while it was positively correlated with Muribaculaceae (R = 0.453, *p <* 0.05). The concentration of TNF-α was positively correlated with Bacteroidota (R = 0.389, *p <* 0.05). The concentration of MAP was negatively correlated with Firmicutes (R = −0.392, *p <* 0.05). The concentration of HP-70 was negatively correlated with Firmicutes (R = −0.479, *p <* 0.01), Patescibacteria (R = −0.446, *p <* 0.05), and *Clostridia_UCG-014* (R = −0.517, *p <* 0.01), while it was positively correlated with Spirochaetota (R = 0.434, *p <* 0.05), Verrucomicrobiota (R = 0.377, *p <* 0.05). The concentration of LPS was negatively correlated with *Succinivibrionaceae_UCG-001* (R = −0.378, *p <* 0.05), while it was positively correlated with Euryarchaeota (R = 0.568, *p <* 0.01), *F082* (R = 0.450, *p <* 0.05), *Methanobrevibacter* (R = 0.568, *p <* 0.01). The concentration of His was positively correlated with *Prevotella* (R = 0.546, *p <* 0.01).

## 4. Discussion

The present results indicate that the inclusion of YC in high-grain diets led to a significant increase in the average daily weight gain of goats. Improvements in ADG have also been reported previously in goats supplemented with YC [39]. Additionally, it decreased F/G and increased the apparent digestibility of crude protein, NDF, and ADF. The relative abundance of rumen microorganisms in goats was changed. Therefore, it could be speculated that YC may enhance goat performance in two ways. First, it may be related to the effect of YC on the rumen internal environment. Second, the active components in YC may improve the body’s immunity, enhancing the goat’s production performance [40,41].

The pH is a reliable indicator of the anaerobic fermentation status of the rumen in ruminants. It is crucial to maintain the pH within the normal range to ensure normal rumen fermentation. Previous studies in cattle have shown that feeding high-grain diets significantly reduces rumen pH and induces rumen acidosis, and YC can decrease the occurrence of rumen acidosis by stabilizing the pH of the rumen through decreasing lactic acid-producing bacteria and increasing lactic acid-utilizing bacteria or rumen protozoa [42,43,44,45,46]. In this trial, it was expected that YC supplementation would prevent rumen acidosis in goats. Rumen pH values in all treatment groups in this trial were higher than 7. Previous studies have also found that rumen pH does not fall below 6 when goats are fed high-grain diets [47]. This phenomenon may be related to differences in rumen organization between cattle and goats. Acetic acid, propionic acid, and butyric acid account for more than 95% of the total volatile fatty acids produced by rumen fermentation [48]. The addition of YC did not significantly affect the content of total volatile fatty acids, acetic acid, propionic acid, and butyric acid in this experiment. The addition of YC to mid-lactation cows also did not have a significant effect on total volatile fatty acids [19,49]. This experiment showed that the addition of YC to the diet tended to decrease the ratio of acetic acid to propionic acid, which may reflect the type of rumen fermentation. Furthermore, the addition of YC to high-grain diets significantly increased MCP content. The results indicated that YC groups promoted rumen nitrogen metabolism, improved ammonia and nitrogen utilization, increased rumen microbial protein production, and facilitated protein deposition compared to the control group. Positive changes in the rumen environment can affect bacterial populations and their fermentation products, leading to improved productivity and nutrient utilization in ruminants [50,51].

Maintaining a micro-ecological balance in the gastrointestinal tract is crucial for the overall health of the organism. The dominant microflora plays a significant role in achieving this balance. An imbalance in the dominant gastrointestinal flora can lead to a dysfunctional microecosystem [52,53]. This study found that the YC groups had a positive effect on bacterial populations, mainly by increasing the relative abundance of Bacteroidota and Fibrobacterota. Supplementation of YC increased the number and activity of fiber-digesting bacteria, resulting in improved fiber digestibility. The relative abundance of *Prevotellaceae_UGG_001* and *Prevotellaceae_UGG_003* among rumen bacteria significantly affected fiber digestibility. Although the relative abundance of these two bacteria did not increase significantly in the YC1 group during this experiment, the fiber digestibility of the YC1 group still showed a significant increase. This may be because fiber degradation is not only caused by the action of one bacterial group alone but by the action of multiple bacterial groups working together [54]. To better understand the effect of YC on the immune performance of goats, the correlation between rumen microbiota and immune index was analyzed in this study. The results showed that there was a negative correlation between the concentration of IL-10 and Bacteroidota, Spirochaetota, and *Succinivibrio*, while there was a positive correlation between the concentration of IL-10 and Firmicutes. The rumen microbiota of ruminants is diverse, with some bacteria considered beneficial, such as *Prevotellaceae_UCG-001* and *Clostridia_UCG-014*. Conversely, other bacteria, such as Streptococcus, may produce harmful substances that cause health problems [55]. YC may improve immunity mainly by altering the composition of rumen microorganisms [56].

Ruminants produce ammonia in their rumen, which is then converted to urea in the liver. The concentration of urea in the blood is highly correlated with the nitrogen cycle and rumen protein degradation and can reflect protein metabolism in the animal [57]. This study found that the urea concentration in the YC2 group was significantly higher than in the CON and YC1 groups. The additional NH_3_-N was used to synthesize bacterial proteins, increasing the efficiency of feed protein utilization by ruminants. Serum TG reflects lipid levels, and its content reflects lipid absorption and metabolism. An increase in serum TG concentration may be due to increased lipase activity and dietary fat utilization by yeast preparations [58]. The serum TG concentration in the YC1 group was significantly higher than that in the control and YC2 groups. YC has the potential to improve the production performance of ruminants by promoting lipid metabolism.

The nutritional modulation of yeast cultures was studied to improve the immunity of ruminants and achieve high production performance. Immunoglobulins are proteins produced by plasma cells that have antibody activity and exert antibacterial and antiviral effects. The IgG, IgM, and IgA are usually tested to reflect the body’s immune status [59]. This experiment’s results showed that the serum IgA and IgG concentrations of the YC1 and YC2 groups were significantly higher than those of the control group. Additionally, the serum IgM concentration of the YC2 group was significantly higher than that of the control group. The addition of yeast cultures to HGD can significantly enhance the immunity of goats by reducing the release of pro-inflammatory factors that impair the integrity of the gastrointestinal tract and the intestinal barrier function [60]. In periparturient cows, the addition of YC to the diet resulted in reduced serum levels of IL-6 and IL-8 and down-regulated the expression level of IL-6 mRNA in the uterus. This study found that the serum levels of IL-1β, IL-6, and TNF-α significantly decreased in the YC1 and YC2 groups. Additionally, the serum level of IL-10 significantly increased in the YC1 group. These results suggest that yeast culture may have anti-inflammatory effects and can reduce the level of inflammatory factors in serum. Wang et al. also found that yeast products have antioxidant, anti-inflammatory, and immunomodulatory effects in vitro [61]. Yeast cell wall polysaccharides, such as β-glucan, mannan, and B-complex vitamins, are the primary nutrient factors that produce these effects. It has been shown that biotin and B-complex vitamins alleviate rumen acidosis in ruminants and are effective in the prevention and treatment of hoof disease in dairy cows [62,63]. The immunomodulatory effects of yeast cultures may be more pronounced when animals are under pathogenic challenges and environmental stress.

Since the composition and mechanism of action of yeast culture products from different companies vary due to differences in production processes and yeast strains, their effects on animal physiological and biochemical indicators are also not consistent. The concentration of triglyceride in the YC1 group was significantly higher than that of the CON and YC2 groups, while the concentration of urea in the YC2 group was significantly higher than that of the CON and YC1 groups. The relative abundance of rumen microorganisms was also differently affected by different types of yeast cultures. At the phylum level, the addition of YC2 to the diet significantly increased the relative abundance of Bacteroidota and Fibrobacterota and significantly decreased Firmicutes compared to the control. At the genus level, the addition of YC1 to the HGD significantly reduced the relative abundance of *Rikenellaceae_RC9_gut_group*, while the addition of YC2 to the HGD significantly increased the relative abundance of *Prevotellaceae_UCG-001*, Fibrobacter, and *Prevotellaceae_UCG-003*. These three bacteria were highly correlated with fiber degradation, suggesting that YC2 may increase the digestibility of cellulose in goats.

## 5. Conclusions

The results observed in this study suggested that supplementation of the two commercial YC in goats’ high-grain diet improved growth performance and improved immunity. The use of yeast cultures also positively influenced rumen fermentation and microbial diversity, particularly by increasing the relative abundance of beneficial bacteria such as Bacteroidota and Fibrobacterota. In the meantime, the microbiota indirectly affects immune function by regulating metabolites. Furthermore, the different types of yeast cultures exerted distinct effects on physiological and biochemical parameters. YC1 significantly increased serum triglyceride levels, while YC2 elevated urea concentrations, reflecting differences in how these cultures modulate lipid and protein metabolism. However, different types of yeast cultures acted by different mechanisms. The selection of a specific yeast culture should be tailored to the production goals and the desired physiological outcomes. Further research is necessary to investigate the long-term effects and underlying mechanisms of different yeast culture formulations in various ruminant species.

## Figures and Tables

**Table 1:** Ingredients and chemical composition of experimental diets (%, dry matter basis).

Item	Group		
	CON	YC1	YC2
Ingredients			
Corn	50.70	50.20	50.20
Soybean meal	10.00	10.00	10.00
DDGS	6.00	6.00	6.00
Alfalfa hay	20.00	20.00	20.00
Corn husk	10.00	10.00	10.00
CaCO_3_	0.80	0.80	0.80
NaCl	0.40	0.40	0.40
Premix	0.55	0.55	0.55
NaHCO_3_	1.00	1.00	1.00
MgO	0.50	0.50	0.50
Antioxidant	0.05	0.05	0.05
Yeast culture	0.00	0.50	0.50
Chemical composition			
Dry matter	89.05	89.05	89.05
Crude protein	14.20	14.20	14.21
Crude ash	6.44	6.44	6.44
Crude fat	4.31	4.31	4.31
Metabolizable energy, M Cal/kg	2.57	2.57	2.57
Neutral detergent fiber	48.05	48.05	48.05
Acid detergent fiber	12.12	12.12	12.12
Calcium	0.56	0.56	0.56
Phosphorus	0.38	0.38	0.38
Concentrate:roughage	80:20	80:20	80:20

The premix provided the following per kg of diet: vitamin A 2200 IU; vitamin D 250 IU; vitamin E 20 IU; Fe 40 mg; Cu 10 mg; Zn 30 mg; Mn 40 mg; I 0.8 mg; Se 0.2 mg; Co 0.11 mg.CON = control group; YC1 = yeast culture1 group; YC2 = yeast culture 2 group.

**Table 2:** Growth performance of different groups in goats.

	Group			*p*-Value
	CON	YC1	YC2	
Initial body weight, kg	25.75 ± 0.42	25.42 ± 0.40	25.72 ± 0.96	0.828
BW at 30 days, kg	30.99 ± 0.23	31.95 ± 0.39	32.20 ± 0.86	0.297
ADG, g/d	174.67 ± 6.82 ^b^	213.33 ± 4.77 ^a^	216.67 ± 9.33 ^a^	<0.001
ADFI, kg/d	1.11 ± 0.02 ^b^	1.15 ± 0.05 ^a^	1.17 ± 0.06 ^a^	0.009
F/G	6.35 ± 0.26 ^a^	5.39 ± 0.21 ^b^	5.41 ± 0.16 ^b^	0.005

CON = control group; YC1 = yeast culture1 group; YC2 = yeast culture 2 group. All values were expressed as means ± standard error. ^a,b^ Means with different superscripts within the same column differ significantly (*p* < 0.05). BW = Body weight. ADG = Average daily gain. ADFI = Average daily feed intake. F/G = Feed intake and weight gain ratio.

**Table 3:** Apparent total tract digestibility of nutrients (%) of different groups in goats.

Item	Group			*p*-Value
	CON	YC1	YC2	
Dry matter	68.07 ± 1.20 ^b^	73.45 ± 1.47 ^a^	72.54 ± 1.55 ^a^	0.002
Crude protein	70.34 ± 2.02 ^b^	72.78 ± 1.22 ^ab^	74.22 ± 1.60 ^a^	0.009
Acid detergent fiber	44.79 ± 1.91 ^b^	52.20 ± 0.84 ^a^	52.43 ± 1.63 ^a^	0.012
Neutral detergent fiber	69.47 ± 1.76 ^b^	74.78 ± 1.16 ^a^	73.48 ± 0.60 ^a^	0.016
Ether extract	71.43 ± 2.27 ^b^	78.38 ± 1.42 ^a^	72.58 ± 2.26 ^b^	0.009

CON = control group; YC1 = yeast culture1 group; YC2 = yeast culture 2 group. All values were expressed as means ± standard error. ^a,b^ Means with different superscripts within the same column differ significantly (*p <* 0.05).

**Table 4:** Jugular vein blood parameters of different groups in goats.

Items	Group			*p*-Value
	CON	YC1	YC2	
Glutamic pyruvic transaminase, ALT, U/L	21.99 ± 1.63	19.93 ± 1.26	20.85 ± 1.63	0.634
Glutamic oxaloacetic transaminase, AST, U/L	66.47 ± 6.26	66.99 ± 6.24	68.43 ± 5.44	0.972
Urea, mmol/L	5.50 ± 0.40 ^b^	6.11 ± 0.45 ^b^	7.61 ± 0.29 ^a^	0.005
Creatinine, CREA, umol/L	55.34 ± 5.34	60.64 ± 5.65	65.98 ± 3.10	0.314
Alkaline phosphatase, ALP, U/L	307.3 ± 87.41	512.10 ± 145.27	348.2 ± 107.02	0.428
Lactate dehydrogenase, LDH, U/L	278.05 ± 18.89	291.41 ± 22.47	296.88 ± 25.35	0.830
Creatine kinase, CK, U/L	109.89 ± 11.22	110.07 ± 14.77	110.67 ± 11.24	0.998
Total protein, TP, g/L	55.34 ± 3.72	54.13 ± 2.65	59.81 ± 2.63	0.393
Albumin, ALB, g/L	27.43 ± 1.72	26.12 ± 1.21	30.35 ± 1.52	0.143
Total cholesterol, TC, mmol/L	1.54 ± 0.12	1.45 ± 0.12	1.68 ± 0.14	0.466
Triglyceride, TG, mmol/L	0.25 ± 0.03 ^b^	0..43 ± 0.07 ^a^	0.28 ± 0.04 ^b^	0.044
Low-density lipoprotein cholesterol, LDL-C, mmol/L	0.54 ± 0.05	0.57 ± 0.05	0.57 ± 0.05	0.904
High-density lipoprotein cholesterol, HDL-C, mmol/L	1.51 ± 0.10 ^ab^	1.33 ± 0.11 ^b^	1.72 ± 0.13 ^a^	0.065
Lipopolysaccharide, LPS, ng/L	249.37 ± 5.89	241.71 ± 7.59	263.88 ± 11.30	0.197
Histamine, HIS, ng/mL	9.59 ± 0.26	9.94 ± 0.21	10.26 ± 0.38	0.286
Interleukin-1β, IL-1β, pg/mL	78.60 ± 10.46 ^a^	36.05 ± 4.17 ^b^	34.07 ± 15.96 ^b^	<0.001
Interleukin-6, IL-6, pg/mL	92.84 ± 8.95 ^a^	39.99 ± 4.53 ^b^	37.69 ± 3.94 ^b^	<0.001
Interleukin-10, IL-10, pg/mL	20.53 ± 1.94 ^b^	41.66 ± 1.48 ^a^	25.70 ± 2.87 ^b^	<0.001
Tumor Necrosis Factor-α, TNF-α, pg/mL	157.76 ± 12.31 ^a^	91.84 ± 5.81 ^b^	97.19 ± 5.21 ^b^	<0.001
Immunoglobulin M, IgM, μg/mL	1733.44 ± 73.71 ^b^	1926.57 ± 87.40 ^ab^	2052.36 ± 80.37 ^a^	0.031
Immunoglobulin A, IgA, μg/mL	152.45 ± 21.18 ^b^	206.60 ± 8.11 ^a^	199.92 ± 11.17 ^a^	0.028
Immunoglobulin G, IgG, mg/mL	5.24 ± 0.43 ^b^	7.01 ± 0.23 ^a^	7.52 ± 0.26 ^a^	<0.001
Major acute phase protein, MAP, mg/L	112.33 ± 9.79	107.59 ± 5.50	97.78 ± 6.79	0.395
Heat shock protein 70, HP-70, pg/mL	375.41 ± 32.46	317.08 ± 24.80	337.57 ± 22.65	0.315

CON = control group; YC1 = yeast culture1 group; YC2 = yeast culture 2 group. All values were expressed as means ± standard error. ^a,b^ Means with different superscripts within the same column differ significantly (*p <* 0.05).

**Table 5:** Rumen fermentation characteristics of different groups in goats.

Item	Group			*p*-Value
	CON	YC1	YC2	
pH	7.05 ± 0.08	7.08 ± 0.06	7.06 ± 0.07	0.655
TVFA, mmol/l	40.36 ± 5.17	39.63 ± 5.49	36.86 ± 3.41	0.871
Acetic acid, mmol/L	25.70 ± 3.54	25.18 ± 23.04	23.04 ± 2.36	0.820
Propionic acid, mmol/L	9.80 ± 1.88	11.25 ± 2.14	10.01 ± 1.08	0.824
Butyric acid, mmol/L	2.88 ± 0.54	1.60 ± 0.26	2.03 ± 0.50	0.136
Isobutyric acid, mmol/L	0.66 ± 0.10	0.55 ± 0.08	0.60 ± 0.07	0.715
Isovaleric acid, mmol/L	0.89 ± 0.11	0.72 ± 0.12	0.81 ± 0.06	0.542
Pentanoic acid, mmol/L	0.49 ± 0.07	0.36 ± 0.08	0.38 ± 0.04	0.306
Acetic acid/Propionic acid	2.94 ± 0.27	2.83 ± 0.60	2.34 ± 0.20	0.565
Microbial protein, μg/L	16.64 ± 2.24 ^b^	28.13 ± 1.90 ^a^	28.60 ± 5.37 ^a^	0.039
NH_3_-N, mg/dl	70.90 ± 13.10	67.09 ± 6.92	60.23 ± 1.66	0.656

CON = control group; YC1 = yeast culture1 group; YC2 = yeast culture 2 group. All values were expressed as means ± standard error. ^a,b^ Means with different superscripts within the same column differ significantly (*p <* 0.05).

**Table 6:** Differences in Alpha-diversity indices.

Item	Group			*p*-Value
	CON	YC1	YC2	
Chao1	802.11 ± 57.60 ^a^	522.66 ± 44.54 ^b^	719.69 ± 47.48 ^a^	0.002
Dominance	0.04 ± 0.00 ^b^	0.10 ± 0.02 ^a^	0.06 ± 0.01 ^ab^	0.051
goods_coverage	0.99 ± 0.00	0.99 ± 0.00	0.99 ± 0.00	0.087
observed_otus	761.6 ± 52.94 ^a^	500.40 ± 43.23 ^b^	677.11 ± 43.42 ^a^	0.002
pielou_e	0.71 ± 0.03 ^a^	0.61 ± 0.03 ^b^	0.68 ± 0.03 ^ab^	0.071
Shannon	6.81 ± 0.31 ^a^	5.49 ± 0.34 ^b^	6.39 ± 0.31 ^ab^	0.020
Simpson	0.96 ± 0.01 ^a^	0.90 ± 0.02 ^b^	0.94 ± 0.01 ^ab^	0.051

CON = control group; YC1 = yeast culture1 group; YC2 = yeast culture 2 group. All values were expressed as means ± standard error. ^a,b^ Means with different superscripts within the same column differ significantly (*p <* 0.05).

**Table 7:** The differential microorganisms in the rumen at phylum and genus levels among groups.

Item	Group			*p*-Value
	CON	YC1	YC2	
**Phylum**				
Bacteroidota	30.27 ± 3.74 ^b^	31.38 ± 4.64 ^b^	52.03 ± 4.25 ^a^	0.002
Firmicutes	36.44 ± 6.44 ^a^	33.78 ± 6.94 ^a^	15.11 ± 1.97 ^b^	0.033
Fibrobacterota	0.24 ± 0.06 ^b^	0.10 ± 0.03 ^b^	0.91 ± 0.21 ^a^	<0.001
**genus**				
*Rikenellaceae_RC9_gut_group*	3.13 ± 0.69 ^a^	0.71 ± 0.23 ^b^	4.17 ± 0.1.18 ^a^	0.012
*Prevotellaceae_UCG-001*	0.96 ± 0.20 ^b^	0.81 ± 0.19 ^b^	2.19 ± 0.45 ^a^	0.006
*Prevotellaceae_UCG-003*	0.41 ± 0.20 ^b^	0.30 ± 0.07 ^b^	1.26 ± 0.28 ^a^	0.004
*Fibrobacter*	0.24 ± 0.06 ^b^	0.10 ± 0.03 ^b^	0.91 ± 0.21 ^a^	<0.001
*Prevotellaceae_UCG-010*	0.45 ± 0.11 ^a^	0.13 ± 0.05 ^b^	0.22 ± 0.04 ^b^	0.018

CON = control group; YC1 = yeast culture1 group; YC2 = yeast culture 2 group. All values were expressed as means ± standard error. ^a,b^ Means with different superscripts within the same column differ significantly (*p <* 0.05). Data represent the relative abundance at greater than 0.1% of the community among the three groups.

## Data Availability

Original data are available from the corresponding author upon reasonable request.
